# Mindfulness-Based Functional Therapy: a preliminary open trial of an integrated model of care for people with persistent low back pain

**DOI:** 10.3389/fpsyg.2014.00839

**Published:** 2014-08-04

**Authors:** Robert Schütze, Helen Slater, Peter O'Sullivan, Jennifer Thornton, Amy Finlay-Jones, Clare S. Rees

**Affiliations:** ^1^School of Psychology and Speech Pathology, Curtin UniversityPerth, WA, Australia; ^2^School of Physiotherapy and Exercise Science, Curtin UniversityPerth, WA, Australia; ^3^Curtin Health Innovation Research Institute, Curtin UniversityPerth, WA, Australia

**Keywords:** low back pain, mindfulness, pain catastrophizing, chronic pain, physiotherapy, clinical psychology, pilot study

## Abstract

**Objectives:** This pilot study investigated the feasibility and clinical utility of implementing a novel, evidence-informed, interdisciplinary group intervention—Mindfulness Based Functional Therapy (MBFT)—for the management of persistent low back pain (LBP) in primary care. MBFT aimed to improve physical and psychological functioning in patients with persistent LBP.

**Design:** A single-group repeated measures design was utilized to gather data about feasibility, effect sizes, clinically significant changes and patient satisfaction.

**Setting:** A community sample of 16 adults (75% female), mean (*SD*) age 47.00 (9.12) years (range 26–65 years), with mean (*SD*) LBP duration of 8.00 (9.00) years participated, using a simulated primary care setting at Curtin University in Australia.

**Intervention:** MBFT is an 8-week group intervention co-facilitated by psychology and physiotherapy disciplines. Content includes: mindfulness meditation training, cognitive-functional physiotherapeutic movement retraining, pain education, and group support.

**Main outcome measures:** Several validated self-report measures were used to assess functional disability, emotional functioning, mindfulness, pain catastrophizing, health-related quality of life at baseline, post-intervention, and 6 months follow-up.

**Results:** Adherence and satisfaction was high, with 85% of participants *highly satisfied* with MBFT. Clinical significance analysis and effect size estimates showed improvements in a number of variables, including pain catastrophizing, physical functioning, role limitations due to physical condition, and depression, although these may have occurred due to non-intervention effects.

**Conclusions:** MBFT is feasible to implement in primary care. Preliminary findings suggest that a randomized controlled trial is warranted to investigate its efficacy in improving physical and emotional functioning in people with disabling persistent LBP.

## Introduction

The current biopsychosocial perspective of low back pain (LBP) acknowledges a dynamic interaction between physical, cognitive, affective, behavioral, genetic, and environmental factors in determining a person's pain experience (Turk and Monarch, 2002; Cassidy et al., 2012). Multidisciplinary management approaches based on this perspective have considerable empirical support (Guzman et al., 2001; Hill et al., 2008, 2010). However, the management of patients with persistent non-specific LBP is unsatisfactory, with evidence indicating that most interventions deliver, at best, only modest benefit (Foster, 2011). Accordingly, the associated health and economic burdens of persistent LBP, at individual and societal levels are significant (Access Economics, 2007; Hoy et al., 2010; Lambeek et al., 2011). Poor outcomes relate to many factors, including the complex and multidimensional nature of persistent pain, which is typically poorly understood by clinicians, policy makers and consumers (Macintyre et al., 2010), and the failure to recognize that subgroups of persistent LBP exist, with these groups potentially requiring different targeted interventions (Hill et al., 2008, 2010; O'Sullivan, 2012).

While the clinical response to the multidimensional nature of pain is best reflected in the focus on Multidisciplinary Pain Management Programs (Airaksinen et al., 2006; Hoffman et al., 2007), few approaches have demonstrated significant longer term efficacy (Turk and Burwinkle, 2005) and most are conducted in tertiary settings rather than in primary care. Furthermore, recent systematic reviews suggest that many multidisciplinary cognitive-behavioral interventions for chronic pain are based on a “pragmatic mix” of components with inadequate theoretical rationale for their inclusion (Eccleston et al., 2009). It is unclear which components of these programs account for improvement and which components may best target specific clinical pain phenotypes.

Based on current persistent LBP subgrouping it is, however, possible to identify a group characterized by higher disability associated with reduced physical and emotional functioning, increased catastrophizing and fear avoidance behaviors, and associated maladaptive movement behaviors (O'Sullivan, 2005, 2012). For this group we proposed an integrated body-mind intervention based on current evidence-based persistent LBP guidelines (Airaksinen et al., 2006) and which would be suitable for implementation in a primary care setting. A targeted intervention is indicated because persistent LBP is associated with pain provocative movements and postural behaviors (O'Sullivan, 2005; Dankaerts et al., 2006) that have been linked to a loss of body awareness and perception (Wand et al., 2010), distorted body maps (Flor et al., 1997), difficulty in integrating body schema with motor processes (Moseley et al., 2008) and a loss of spine repositioning sense (O'Sullivan et al., 2003).

Furthermore, mindfulness, which involves non-evaluative, present-focused awareness of physical and psychological experience (Kabat-Zinn, 1990), may also help to modulate the avoidance behaviors associated with persistent LBP that can contribute to the fear-avoidance cycle. As catastrophizing is arguably the strongest psychological predictor of long-term pain and disability in chronic pain due to its relationship to fear and avoidance (Picavet, 2002; Sullivan et al., 2004; Severeijns et al., 2005; Leeuw et al., 2007), mindfulness training could also be an effective way to deliver the benefits intended in traditional psychological pain management approaches. Mindfulness is associated with reduced pain catastrophizing (Gardner-Nix et al., 2008; Schütze et al., 2010) and there is mounting evidence that mindfulness interventions are themselves effective in easing the burden of chronic pain (Teixeira, 2008; Chiesa and Serretti, 2011; Brown, 2013).

With this in mind, the present pilot study developed and tested an integrated Mindfulness-Based Functional Therapy (MBFT) intervention, which combined mindfulness meditation, physiotherapy movement retraining, and psycho-education in an 8-week group program. The study aimed to test whether this intervention was: (i) feasible and acceptable when implemented in a primary care setting and; (ii) associated with sufficient improvements in physical and psychological functioning to warrant further research using a more robust study design with a bigger sample.

## Materials and methods

### Study design

This pilot study utilized a pre-experimental single group, repeated measures design in order to gather data about effect sizes, clinically significant changes, and patient satisfaction. Since the trial was designed to test the MBFT protocol and the feasibility of undertaking a larger, more comprehensive trial, a control group was not used.

### Inclusion/exclusion criteria

Inclusion criteria required that participants be aged between 18 and 65 years of age and have persistent LBP, defined in accordance with the International Association for the Study of Pain's (IASP) Task Force on Taxonomy as “pain that continues beyond the usual course of healing, usually taken to mean continuous or intermittent pain for at least three months” (Merskey and Bogduk, 1994). A cut-off score of 85 on the Orebro Musculoskeletal Pain Questionnaire (OMPQ) was used to exclude those with lower functional impairment and disability (Hill et al., 2010). Additional exclusion criteria included: (1) current use of medications containing an daily oral morphine equivalent dose of 100 mg; (2) predominant neuropathic pain conditions including dominant lower leg radicular pain with clinical signs of neuropathic pain as defined by the IASP (Treede et al., 2008); (3) low back spinal surgery in the past 12 months; (4) current substance abuse or dependence; (5) high risk of suicide; (6) active psychotic conditions, and (7) limited English language fluency.

### Recruitment

Participants were recruited from the general population through advertisements in health clinics and hospitals in metropolitan Perth, Australia. A convenience sample of physiotherapists, psychologists, and pain specialists in the Perth metropolitan area were also contacted and invited to provide referrals for the study cohort.

### Procedure

This study was approved by the Curtin University Human Research Ethics Committee (approval HR72/2009) and adhered to the Declaration of Helsinki. Participants registered their interest in the study by contacting the Curtin University Psychology Clinic. Participants were then telephone-screened via Clinical Psychology (RS) prior to recruitment. Participants who met the inclusion criteria were then further individually screened by a senior specialist musculoskeletal physiotherapist (HS) experienced in pain management, who made a clinical judgment about their likely ability to safely complete the MBFT intervention (i.e., no specific neurological deficit or other serious pathology indicating a precaution or contraindication to participating). After being assessed and accepted for inclusion into the study, participants completed a pre-intervention questionnaire battery (outlined below) on day 1 of the intervention, and this battery of measures was completed again immediately at post-intervention (on the last day of the intervention) and at 6 months post-intervention.

### Intervention

The MBFT group intervention was developed specifically for this study, as outlined in the *Mindfulness-Based Functional Therapy Facilitator Manual* (unpublished document available on request). The intervention was comprised of an integrated weekly 2-h group session run over eight consecutive weeks and involved: group discussion; homework review; psycho-education; and mindfulness meditation aligned with cognitive behavioral physiotherapy re-education of normal relaxed movement and postures. All MBFT sessions were co-facilitated by the same clinical psychology and physiotherapy team (RS and HS). For pragmatic reasons, there were two intervention groups, with 10 participants in the first group and 6 in the second. While taking part in the intervention, participants were instructed to continue their usual activities and their usual medical care, including medications.

The mindfulness component of MBFT was adapted from the well-known Mindfulness-Based Stress Reduction (MBSR; Kabat-Zinn, 1990) and Mindfulness-Based Cognitive Therapy for Depression (MBCT; Segal et al., 2002) protocols. This involved in-session meditation exercises (for example, body scan meditation; awareness of breathing meditation; walking meditation; awareness of thoughts meditation), reflection and discussion on the meditation experience, psycho-education about the mindfulness meditation exercises, and daily home mindfulness practice using audio recordings of the guided meditations used in MBSR. The in-session mindfulness exercises were adapted specifically for a persistent LBP population, with tailored instructions on working with pain and the associated cognitions and affects. The physiotherapy component targeted basic functional movements that are commonly compromised in patients with persistent LBP, and which can contribute to the maintenance of pain (O'Sullivan, 2005; Dankaerts et al., 2006, 2009). The aim was to train participants to practice mindfulness in functional daily movements and postures, and using a graded approach, to progressively moderate persistent maladaptive protective or avoidant movement behaviors (O'Sullivan, 2005; Dankaerts et al., 2006, 2009). These functional postures and exercises included: body awareness of breathing; movement control involving progressing from non-weight bearing to weight bearing (e.g., rolling, sitting, bending, lifting, standing, and walking); transitioning between postures (e.g., sitting to standing); and targeting specific individual pain provocative tasks identified by the participant. An emphasis was placed on relaxed breathing and gentle, appropriately graduated movements avoiding excessive abdominal bracing, breath-holding, or inappropriate strategies such as hand support to get from sitting to standing. Graduated mindful virtual and real rehearsal of previously avoided, or pain provocative movements and postures, was encouraged. The aim of practizing these functional movements as virtual movements was to further reinforce non-provocative sensorimotor integration (Flor and Diers, 2009). A graduated cardiovascular program which emphasized time-contingent (McCracken and Samuel, 2007) rather than pain-contingent (Abbott et al., 2010) pacing was also implemented.

Movement re-training exercises were blended into each session to complement the mindfulness exercises (e.g., targeting walking in the session where walking meditation was introduced). The intention of this approach was thereby to integrate the psychology and physiotherapy components of this intervention, rather than present each as separate components, by separate disciplines as appears common in many multidisciplinary pain rehabilitation programs (Eccleston et al., 2009). The educational component of MBFT used didactic and interactive delivery of current evidence-based information about pain management, covering: pain neurophysiology, central nervous system sensitization, the biopsychosocial model of pain; treatments for spinal pain; effective use of medications; importance of return to normal movement/exercise in modulating pain; the fear-avoidance model of chronic pain; and the role of stress and negative affect in maintaining pain.

### Measures

The following measures were chosen to align with current IMMPACT (Dworkin et al., 2008) recommendations for the clinical importance of treatment outcomes in chronic pain clinical trials.

The Örebro Musculoskeletal Pain Questionnaire (OMPQ; Linton, 1999) is a 25-item screening tool used to assess the risk of future disability in pain patients. It includes items covering physical functioning, emotional functioning, and pain intensity. The OMPQ has been found to have test-retest reliability of 0.83 and to correctly identify 80% of acute pain patients who would not return to work (Linton and Boersma, 2003; Linton, 2005). The OMPQ was used in screening and questions 9-10, which are 10-point numerical rating scales (NRS) of pain, were averaged to form a measure of pain intensity.

The Oswestry Disability Questionnaire (ODQ; Fairbank et al., 1980) is a 10-item measure of low-back related functional disability. The ODQ has been widely validated, has a test-retest reliability of 0.83 to 0.99 and an internal consistency coefficient of 0.71 to 0.87 (Fairbank and Pynsent, 2000).

The short form of the Depression Anxiety Stress Scales (DASS-21; Lovibond and Lovibond, 1995) is a 21-item measure of emotional functioning, with 3 subscales. The DASS-21 has been shown to have high internal consistency, with Cronbach's alphas of 0.94 for Depression, 0.87 for Anxiety, and 0.91 for Stress (Antony et al., 1998; Henry and Crawford, 2005).

The Mindful Attention Awareness Scale (MAAS; Brown and Ryan, 2003) is a 15-item measure of present-moment awareness of actions, interpersonal communication, thoughts, emotions, and physical states. The MAAS has been found to have good convergent and discriminant validity, excellent test-retest reliability (*r* = 0.81, *p* < 0.0001), and good internal consistency, with a coefficient alpha of 0.87 (Brown and Ryan, 2003).

The Pain Catastrophizing Scale (PCS: Sullivan et al., 1995) is a 13-item self-report measure of the degree to which people experiencing pain adopt exaggerated negative interpretations of their pain. The PCS has good criterion-related validity and excellent internal consistency, with a reliability coefficient of 0.92 (Osman et al., 2000).

The Chronic Pain Acceptance Questionnaire (CPAQ) is a 20-item instrument that measures acceptance of pain. The CPAQ has been found to predict patient functioning and is internally consistent, with a coefficient alpha of 0.78–0.84 (McCracken et al., 2004).

The Rand 36-Item Health Survey (SF-36; Ware and Sherbourne, 1992) is a 36-item measure of health status and health-related quality of life. The SF-36 is comprised of eight subscales: (1) physical functioning, (2) role limitations due to physical health, (3) role limitations due to emotional problems, (4) energy/fatigue, (5) emotional well-being, (6) social functioning, (7) pain, and (8) general health. Subscales have internal consistency coefficients of 0.83–0.91 (Davidson and Keating, 2002). The pain subscale was not used as a measure of pain intensity in this study because it takes into account both intensity and interference. Since emotional functioning was measured by the DASS, the emotional well-being subscale was not included in analysis.

The Client Satisfaction Questionnaire (CSQ; Larsen et al., 1979) is an 18-item service evaluation measure that has a high degree of internal consistency (0.91), and is substantially correlated with service utilization and degree of client-reported change (Larsen et al., 1979).

### Statistical analysis

Data analysis was carried out in the following order: (1) data screening; (2) computing descriptive statistics for all variables using PASW Statistics 18.0 for Macintosh (Spss Inc, 2010); (3) testing for group changes between pre- and post-test, and between pre-test and 6-month follow-up, using a series of paired *t*-tests in PASW Statistics 18.0; (4) calculating effect sizes for pre- to post- intervention and pre-intervention to follow-up changes using Cohen's *d* statistic; (5) conducting power analyses for each test using G*Power (Erdfelder et al., 1996); and (6) testing the clinical significance of the study outcomes using the methodology outlined by Jacobson and Truax (1991). This latter formula defines clinically significant change as movement to within two standard deviations of the mean of the normal population on a particular variable. Normative data from the functional population was used to calculate cut-off scores to determine whether participants were in the normal population at post-treatment and follow-up (Lovibond and Lovibond, 1995; Bowling et al., 1999; Fairbank and Pynsent, 2000; Van Damme et al., 2002). This approach to clinical significance testing is more stringent than several other methods of measuring minimal clinically important differences (Dworkin et al., 2008).

## Results

### Participant inclusion and attrition

A total of 23 people were referred and screened for the study, 7 of which did not meet the inclusion criteria. Sixteen people (12 females and 4 males) participated in the study. Two participants dropped out and, due to missing values, their data were excluded. A further two participants' data were excluded due to a large number of missing values. Pre- and post-intervention data were available for 12 participants. Two participants did not return follow-up questionnaires, leaving 10 participants' data in the follow-up analyses.

### Characteristics

Descriptive statistics for outcome measures at pre-test, post-test, and follow-up are presented in Table 1.

**Table 1:** **Mean values (standard deviations) for outcome measures at baseline, post- intervention and at follow-up**.

**Measure**	**Variable**	**Pre-treatment**	**Post-treatment**	**6-month follow-up**
OMPQ	Pain intensity	4.83 (1.01)	4.29 (0.84)	4.10 (1.52)
ODQ	Functional disability	30.50 (11.41)	27.07 (11.28)	27.10 (11.40)
PCS	Catastrophizing	18.66 (10.55)	7.83 (3.93)	9.10 (6.64)
MAAS	Mindfulness	3.48 (0.87)	3.88 (0.97)	4.31 (0.82)
CPAQ	Pain acceptance	60.33 (14.22)	72.58 (16.86)	81.50 (20.74)
DASS	Depression	9.00 (5.88)	6.67 (5.93)	4.40 (2.63)
	Anxiety	5.67 (5.90)	4.83 (5.62)	3.01 (0.95)
	Stress	16.67 (9.08)	11.33 (9.12)	8.60 (5.25)
SF-36	Physical functioning	50.42 (19.24)	55.42 (22.20)	63.50 (20.28)
	Role limit.—physical	12.50 (16.86)	43.75 (37.12)	50.00 (40.82)
	Energy	34.58 (17.90)	53.33 (12.67)	49.50 (27.63)
	Social functioning	45.84 (17.13)	59.38 (28.76)	70.00 (30.16)
	General health	52.08 (14.84)	63.33 (20.15)	62.00 (22.01)

n = 12 for pre-treatment and post-treatment values, n = 10 for follow-up values. OMPQ indicates Örebro Musculoskeletal Pain Questionnaire; ODQ, Oswestry Disability Questionnaire; PCS, Pain Catastrophizing Scale; MAAS, Mindful Attention and Awareness Scale; CPAQ, Chronic Pain Acceptance Questionnaire DASS, Depression, Anxiety, Stress Scales; SF-36, Rand 36-Item Health Survey.

### Group changes over time

Prior to testing for group changes in outcome variables over time, data were screened for suitability of performing paired samples *t*-tests. The majority of variables met assumptions of normality, despite the Shapiro-Wilk test of normality being violated in five of the variables. A visual inspection of histograms indicated four of these were either normally distributed or were not identified as having a significant amount (greater than 0.01) of skewness or kurtosis. Given that the *t*-statistic is relatively robust against these minor violations, the data were deemed appropriate for analysis (Tabachnick and Fidell, 2007).

Table 2 summarizes the changes in outcome measures between pre- and post-intervention, and between pre-intervention and 6 month follow-up. Statistically significant improvements in 9 of the 13 variables (functional disability, catastrophizing, stress, pain acceptance, physical functioning, role limitations due to physical condition, energy, social functioning, and general health) were evident comparing pre- and post-intervention with alpha 0.05. At the Bonferroni-corrected alpha level of 0.004, energy and catastrophizing were the only variables showing significant pre-post changes, with pain acceptance and general health approaching significance (*p* = 0.006 and *p* = 0.005, respectively). Between pre-intervention and follow-up there were significant changes in 10 of the 13 variables (catastrophizing, mindfulness, pain acceptance, depression, stress, physical functioning, role limitations due to physical condition, energy, social functioning and general health) with alpha 0.05. At the more conservative Bonferroni-corrected alpha level (0.004), physical functioning and social functioning were significant, while pain acceptance, depression and mindfulness were approaching significance (*p* = 0.004, 0.006, and 0.008, respectively).

**Table 2:** **Paired *t*-tests for changes in outcome measures comparing baseline and 2 post-intervention time points**.

**Measure**	**Variable**	**Between pre-treatment and post-treatment**			**Between pre-treatment and 6-month follow-up**		
		**(*n* = 12)**			**(*n* = 10)**		
		***t***	***df***	**Sig**.	***t***	***df***	**Sig**.
OMPQ	Pain intensity	1.52	11	0.078	1.60	9	0.072
ODQ	Functional disability	2.28	11	0.022^*^	1.63	9	0.072
PCS	Catastrophizing	3.77	11	0.002^***^	2.14	9	0.030^*^
MAAS	Mindfulness	−1.52	11	0.800	−2.94	9	0.008^**^
CPAQ	Pain acceptance	−2.96	11	0.006^**^	−3.42	9	0.004^**^
DASS	Depression	1.08	11	0.152	3.16	9	0.006^**^
	Anxiety	0.49	11	0.316	1.71	9	0.061
	Stress	1.96	11	0.038^*^	2.44	9	0.018^*^
SF-36	Physical functioning	−1.86	11	0.044^*^	−4.04	9	0.002^***^
	Role limit.—physical	−2.70	11	0.010^*^	−2.81	9	0.010^*^
	Energy	−4.22	11	0.000^***^	−2.41	9	0.020^*^
	Social functioning	−1.95	11	0.039^*^	−3.58	9	0.003^***^
	General health	−3.08	11	0.005^**^	−2.24	9	0.026^*^

* p < 0.05 (one-tail).

** p < 0.01 (one-tail).

*** p < 0.004 (one-tail); this is the Bonferroni-corrected individual alpha level required to maintain experimentwise alpha at p < 0.05. OMPQ indicates Örebro Musculoskeletal Pain Questionnaire; ODQ, Oswestry Disability Questionnaire; PCS, Pain Catastrophizing Scale; MAAS, Mindful Attention and Awareness Scale; CPAQ, Chronic Pain Acceptance Questionnaire DASS, Depression, Anxiety, Stress Scales; SF-36, Rand 36-Item Health Survey.

### Effect size

Between pre- and post-intervention, large effect sizes were obtained for pain catastrophizing, role limitations due to physical condition and energy, as shown in Table 3. Between pre-intervention and 6-month follow-up, large effect sizes were evident for pain catastrophizing, mindfulness, pain acceptance, depression, stress, role limitations due to physical condition, and social functioning.

**Table 3:** **Effect Sizes and *post-hoc* power analyses for changes in outcome measures**.

**Measure**	**Variable**	**Pre- to post- treatment**			**Pre-treatment to 6-month follow-up**		
		**(*n* = 12)**			**(*n* = 10)**		
		**Cohen's *d* (95% CI)**	**Effect size**	***Post-hoc* power**	**Cohen's *d* (95% CI)**	**Effect Size**	***Post-hoc* power**
OMPQ	Pain intensity	0.58 (−0.25–1.38)	Medium	0.41	0.58 (−0.30–1.41)	Medium	0.42
ODQ	Functional disability	0.30 (−0.51–1.10)	Small	0.69	0.30 (−0.56–1.13)	Small	0.36
PCS	Catastrophizing	1.36 (0.43–2.20)	Large^*^	0.99	1.06 (0.13–1.91)	Large^*^	0.78
MAAS	Mindfulness	−0.43 (−1.23–0.39)	Small	0.41	−0.98 (−1.83 to −0.06)	Large^*^	0.92
CPAQ	Pain acceptance	−0.79 (−1.59–0.07)	Medium	0.88	−1.21 (−2.07 to −0.26)	Large^*^	0.95
DASS	Depression	0.39 (−0.43–1.19)	Small	0.26	0.98 (0.06–1.82)	Large^*^	0.98
	Anxiety	0.15 (−0.66–0.94)	Small	0.12	0.60 (−0.28–1.44)	Medium	0.60
	Stress	0.59 (−0.25–1.38)	Medium	0.57	1.06 (0.13–1.91)	Large^*^	0.83
SF-36	Physical functioning	−0.24 (−1.04–0.57)	Small	0.58	−0.66 (−1.50–0.22)	Medium	0.91
	Role limitations—physical	−1.08 (−1.90 to −0.19)	Large^*^	0.86	−1.25 (−2.11 to −0.29)	Large^*^	0.93
	Energy	−1.21 (−2.03 to −0.30)	Large^*^	0.99	−0.65 (−1.49–0.23)	Medium	0.85
	Social functioning	−0.57 (−1.37–0.26)	Medium	0.64	−1.01 (−1.86 to −0.09)	Large^*^	0.98
	General health	−0.64 (−1.43 to 0.20)	Medium	0.93	−0.54 (−1.37–0.33)	Medium	0.74

* Indicates effect size classified as large according to Cohen's (1988) criteria. OMPQ indicates Örebro Musculoskeletal Pain Questionnaire; ODQ, Oswestry Disability Questionnaire; PCS, Pain Catastrophizing Scale; MAAS, Mindful Attention and Awareness Scale; CPAQ, Chronic Pain Acceptance Questionnaire DASS, Depression, Anxiety, Stress Scales; SF-36, Rand 36-Item Health Survey.

### Clinical significance

Table 4 summarizes the results of reliable change and clinically significant change analyses for pre-post intervention and pre-intervention to follow-up changes. Immediately after the intervention, a majority of participants failed to show reliable change on measures of functional disability, mindfulness, acceptance, anxiety, physical functioning, energy, social functioning and general health. However, half the sample had clinically significant change and were therefore classified as *recovered* on catastrophizing, depression, and role limitations due to physical condition. At follow-up, the majority of participants failed to demonstrate reliable change on functional disability, catastrophizing, mindfulness, depression, anxiety, social functioning and general health. However, the vast majority of participants were recovered on physical functioning and role limitations due to physical condition, while half were recovered on stress and energy. A further 70% of participants had reliable change on pain intensity and were therefore classified as *improved*.

**Table 4:** **Reliable change and clinically significant change as outcome variables**.

**Measure**	**Variable**	**Between pre-treatment and post-treatment (*n* = 12)**				**Between pre-treatment and 6-month follow-up**			
		**Det**.	**Unc**	**Imp**.	**Rec**.	**Det**.	**Unc**.	**Imp**.	**Rec**.
OMPQ	Pain intensity	1 (8%)	4 (33%)	7 (58%)	0	1 (10%)	2 (20%)	7 (70%)	0
ODQ	Functional disability	0	10 (83%)	1 (8%)	1 (8%)	0	8 (80%)	1 (10%)	1 (10%)
PCS	Catastrophizing	0	6 (50%)	0	6 (50%)	0	8 (80%)	0	2 (20%)
MAAS	Mindfulness	0	9 (75%)	3 (25%)	0	0	6 (60%)	4 (40%)	0
CPAQ	Pain acceptance	0	9 (75%)	3 (25%)	0	0	4 (40%)	6 (60%)	0
DASS	Depression	0	6 (50%)	0	6 (50%)	0	6 (60%)	0	4 (40%)
	Anxiety	0	10 (83%)	0	2 (17%)	0	8 (80%)	0	2 (20%)
	Stress	0	6 (50%)	1 (%)	5 (42%)	0	5 (50%)	0	5 (50%)
SF-36	Physical functioning	0	10 (83%)	0	2 (17%)	0	2 (20%)	0	8 (80%)
	Role limit.—Physical	1 (8%)	5 (42%)	0	6 (50%)	2 (20%)	1 (10%)	0	7 (70%)
	Energy	0	7 (58%)	0	5 (42%)	1 (10%)	4 (40%)	0	5 (50%)
	Social functioning	0	9 (75%)	0	3 (25%)	0	6 (60%)	0	4 (40%)
	General health	0	9 (75%)	0	3 (25%)	0	9 (90%)	0	1 (10%)

*Det., indicates deteriorated; Unc., unchanged; Imp., improved; Rec., recovered. Using Jacobsen and Truax's ^57^ classification system, positive reliable change is deemed improved, positive reliable changed within two standard deviations of the population mean is recovered (and also improved). Changes below the threshold of the reliable change index (RCI) are unchanged, while negative reliable change is classed as deteriorated. Pain intensity reductions of 1–3 points on the 0–10 OMPQ numerical rating scale were classified as improved, while improvements of at least 4 points were recovered ^39^. Pain increases of at least one point were classified as deteriorated. Reliable change (improved or deteriorated) but not clinical significance (recovered) was calculated for Mindfulness (MAAS) and Pain Acceptance (CPAQ) since they are process variables rather than pain outcome variables*.

### Adherence and satisfaction

Of the 16 participants who entered the study 2 dropped out, one for logistic reasons and one due to a lack of self-perceived benefit. The overall attendance rate of sessions (including dropouts) was 87%. Insufficient data was returned to report on homework adherence. Scores on the Client Satisfaction Questionnaire (CSQ) ranged from 24 to 32, where 32 was also the maximum possible score. The mean score was 30.15 (*SD* = 2.85). All of the participants rated the intervention as satisfactory or higher, with the vast majority (85%) rating the intervention as highly satisfactory. Qualitative data recorded on the CSQ were also largely positive, with comments such as: “I was very happy with the program and found it helpful, many thanks”; and “I am very grateful firstly for this program being recommended to me and for meeting the facilitators who are so dedicated to their professions and to sincerely caring for our wellbeing.”

## Discussion

This study investigated the feasibility and clinical utility of implementing a novel, evidence-informed, interdisciplinary group intervention for the management of persistent LBP. Overall, the MBFT intervention was feasible to implement in a primary care setting, with a high level of adherence and satisfaction recorded. Various analyses, including tests of statistically and clinically significant change, also suggest that participants experienced a range of health improvements over the course of this intervention, although the lack of a control group in this small pilot study means we cannot definitively attribute these changes to the intervention. We observed statistically significant improvement in the majority of outcome measures at both post-test and follow-up. However, in more stringent tests of clinical significance a number of outcomes remained unchanged, while a few notable variables met criteria for recovery. Overall, the high level of patient satisfaction and pattern of improvements in various pain-related outcomes suggests that there is merit in further investigating MBFT using a more robust design. In particular, present findings suggest there is value in exploring how MBFT may improve outcomes such as pain catastrophizing, energy, physical functioning and role limitations.

Surprisingly, despite statistically significant change at post-test, there was little clinically significant improvement in one of our key outcomes of interest—functional disability, as measured by the Oswestry Disability Index. However, this may be related to the responsiveness of this tool, which has been found to be less sensitive to change than some other back disability measures (Lauridsen et al., 2006). By contrast, on the SF-36 large improvements were observed in self-reported physical functioning and role limitations due to physical health, which are also indices of pain-related functional disability. At follow-up these improvements were clinically significant for the vast majority of participants on both variables (80% for physical functioning and 70% for role limitations), thereby meeting relatively stringent criteria for recovery (Jacobson and Truax, 1991). Given the clinical profile of our persistent LBP group (higher disability and substantial pain duration), it is unlikely that these improvements reflect natural processes such as a regression to the mean. This is promising because, given the often long-term and intractable nature of persistent pain, it is important for people living this experience to be able to engage constructively in daily activities, even if pain intensity is not reduced. This was a major focus of the MBFT protocol and improved functioning is central to IMMPACT (Dworkin et al., 2008) recommendations for clinically important treatment outcomes in chronic pain trials. However, these possible intervention effects can only be confirmed with a larger controlled trial.

We observed robust improvements in catastrophizing among our sample, with half meeting criteria for recovery at post-test, while group analyses showed large effect sizes and statistically significant change at a conservative alpha level. This is consistent with previous research showing significant reductions in catastrophizing following multidisciplinary pain interventions, as well as following mindfulness interventions (Gardner-Nix et al., 2008; Cassidy et al., 2012). Furthermore, a large body of evidence linking catastrophizing to fear-avoidance behavior (Leeuw et al., 2007) suggests that this reduction in catastrophizing would translate to greater activity engagement as these activities are possibly interpreted in less threatening ways (Vowles and McCracken, 2008).

MBFT aimed to use mindfulness meditation to attenuate the effect of pain-related threat cognitions by encouraging metacognitive changes, allowing such catastrophic cognitions to be viewed as mental events rather than facts (Hayes et al., 1999; Segal et al., 2002; Wells, 2005). This metacognitive focus is central to so-called third wave psychological approaches to managing depression and anxiety (Öst, 2008) and was a central feature of the MBFT protocol. Data from a recent controlled trial suggests that mindfulness training is as effective as a traditional multidisciplinary pain intervention in reducing pain and pain-related distress in chronic pain patients (Wong et al., 2011). In our study, the improvements in mindfulness, pain-acceptance, and catastrophizing are consistent with previous findings showing a reciprocal inverse relationship between mindfulness and catastrophizing (Gardner-Nix et al., 2008; Schütze et al., 2010; Cassidy et al., 2012). In line with the fear-avoidance model of pain (Vlaeyen and Linton, 2000; Leeuw et al., 2007), we speculate that being more mindful (and therefore less catastrophic) facilitates improved physical functioning by enabling people to engage more confidently (less fearfully) in movement-based functional activities (Smeets et al., 2006). It is also possible that a reciprocal effect emerges, whereby the adoption of graded physical activity without protective behaviors reassures patients that movement is not threatening, thereby lowering catastrophizing.

These speculations should be explored in future studies. A well-designed randomized controlled trial of MBFT, tested against an effective active control such as cognitive behavior therapy, is the first logical step toward investigating efficacy. This pilot data suggests that important domains to measure include pain catastrophizing, mindfulness, physical functioning, functional disability, pain intensity, energy and depression. Catastrophizing and mindfulness may be important moderator/mediator variables that are used to study treatment mechanisms. Recent reviews of psychological treatments for chronic pain have stressed the need to investigate not just efficacy but mechanisms of change in order to improve these therapies (Day et al., 2012; Skinner et al., 2012). A strength of the current study is that it proposes several change mechanisms, in particular that improvements in psychological distress (e.g., catastrophizing, depression, stress) are mediated/moderated by improvements in mindfulness. Similarly, improvements in physical function are proposed to be mediated/moderated by changes in movement patterns.

Although we observed mixed results in this pilot study, with several outcome domains remaining unchanged, there were enough improvements in important pain outcomes to warrant further investigation. This, along with the high levels of participant satisfaction and adherence, suggest further research into this integrated, whole person approach to the co-management of persistent LBP is justified.

### Conflict of interest statement

The authors declare that the research was conducted in the absence of any commercial or financial relationships that could be construed as a potential conflict of interest.
